# Pheochromocytoma of the urinary bladder: a case report

**DOI:** 10.4076/1757-1626-2-8585

**Published:** 2009-07-22

**Authors:** Mohammed Fadl Tazi, Youness Ahallal, Elmehdi Tazi, Amal Benlemlih, Leila Chbani, Afaf Amarti, Mohammed Jamal EL Fassi, Moulay Hassan Farih

**Affiliations:** 1 Department of UrologyCHU Hassan II, 30000, FezMorocco; 2 National Institute of OncologyRabatMorocco; 3 Department of HistopathologyCHU Hassan II, 30000, FezMorocco

## Abstract

Urinary bladder pheochromocytoma is rare. From a case report of unsuspected pheochromocytoma and literature review, the authors develop a diagnostic and therapeutic algorithm for the management of this ectopic pheochromocytoma localization.

## Introduction

Pheochromocytoma is a neuroendocrine tumour that usually develops ahead of the chromaffin cells of the adrenal medulla. The Pheochromocytoma of the urinary bladder accounts for less than 1% of the pheochromocytomas and less than 0.06% of the bladder tumors [1]. The usual signs are haematuria, hypertension during micturition together with generalized symptoms due to raised catecholamines (headache, blurred vision, heart palpitation, flushing). However, 27% of pheochromocytomas of the urinary bladder do not feature any hormonal activity [2].

Following an observation of pheochromocytoma of the urinary bladder, this paper aims at reminding the clinical, therapeutic and histological features of this rare tumour.

## Case presentation

**Figure 1.**
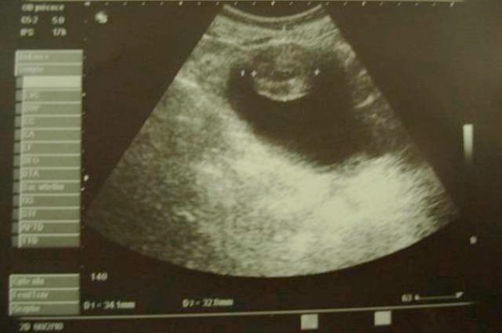
Ultrasonography showing a heterogeneous mass located in the bladder dome.

**Figure 2.**
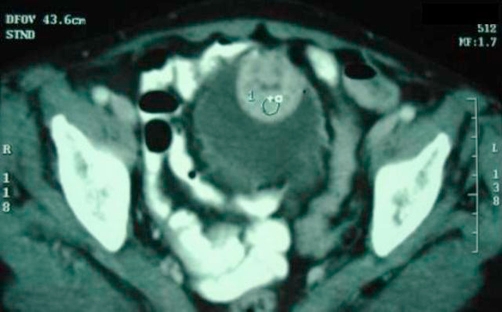
CT showing tumour located in the anterior front of the bladder with well defined bounds.

A 55 year-old Moroccan housewife presented with a 6 months history of haematuria associated with anemia. The physical and paraclinic examinations were normal, her blood pressure showed a 120/80 mmhg. Ultrasonography revealed a 4 cm size heterogeneous mass located in the bladder dome (Figure 1). Computed tomography of the abdomen showed a well defined 3.5 cm size tumour located in the anterior front of the bladder, with a heterogeneous increase in density after injection of iodine-containing contrast solution (Figure 2).

**Figure 3.**
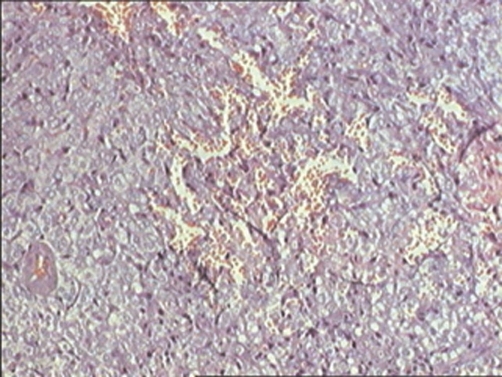
Tumourous proliferation composed of small cells associated to endocrine visualization. (HES x 10).

**Figure 4.**
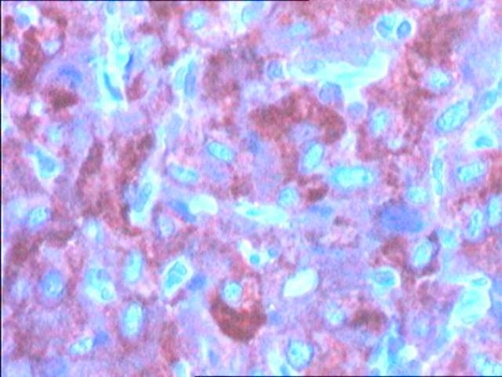
Tumourous cells showing a positive immunostaining for synaptophysin. (HES x 40).

**Figure 5.**
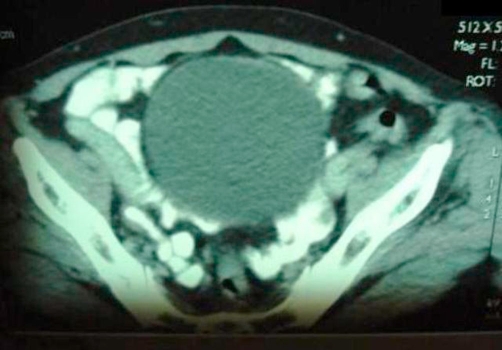
CT showing a greater thickness of the anterior front of the bladder increasing its density after the injection of iodine-containing contrast solution.

Cystoscopy demonstrated a smooth tumour in the wall of the bladder with normal mucosa and transurethral resection provided tissue material for histological examination that suggested a bladder paraganglioma (Figures 3, 4). Dosage of metanephrine and normetanephrine in 24-hour urine was anomaly free computed tomography performed one month later showed a greater thickness of the anterior front of the bladder, with an increase in density after the injection of iodine-containing contrast solution (Figure 5). Partial cystectomy was therefore performed. The postoperative course was uneventful. Histological examination of the tumor disclosed typical findings of pheochromocytoma.

## Discussion

Pheochromocytoma develops from the germinal cells of neural crest, and is usually an adrenal tumor. The Pheochromocytoma of the bladder was first described by Zimmermann in 1953 [3], and more than 70 cases have been spotted since then. The paraganglioma of the bladder is very rare and represents less than 6% of the whole range of paragangliomas and less than 1% of the bladder tumors [4]. The paraganglioma of the bladder is usually a unique tumor with a submucous development [1]. The average age of the patients are relatively low, nearby 40 years, with a range going from 10 to 78 years, with high frequency during the adolescence (17% of the patients hit were less than 15) and the thirties. There is no sex predominance in opposition to the adrenal pheochromocytoma that is affecting the female gender in bigger proportion [5].

Typical symptoms are sharp headaches, hypertension, palpitation and sweating. Increased catecholamine release in association with bladder contraction during micturition can also provoke faintness immediately after bladder voiding. Hypertensive crises may be triggered by micturition, defecation, sexual activity, ejaculation or bladder instrumentation. These evocative symptoms are observed in 65% of the cases [4]. Haematuria can suggest the diagnosis as 65% of the patients were concerned. Paroxysmal hypertension was reported in 65% of the cases [4,5]. The clinical examination can unveil on a hypogastric mass with the size of a nutshell. The above mentioned clinical table recommends the measurement of catecholamines and catecholamine metabolites (metanephrine and normetanephrine) in plasma and 24-hour urine samples. Signs of hormonal activity are displayed in 83% of the bladder pheochromocytomas.

In a recent study on patients affected by extra-adrenal pheochromocytomas, the urinary metanephrine rate was high in 88% of the cases (greater than 0.9 mg/24 h) [6]. In 71% of the cases, the vanyl-mandelic acid (VMA) measurement was positive (greater than 6.8 mg/24 h) and a high concentration of plasmatic catecholamines was discovered in 88% of the patients. However, our patient had a normal urinary catecholamines measurement. The VMA dosage has not been performed as the secreting activity of the tumor occurs only during the contractile phase of the detrusor. It can persist only 5 minutes afterwards, which questions the reliability of the 24 hours measurement [7,8]. Ultrasonography and computed tomography (CT) maintain the tissue nature of the tumor, with an increasing density after iodine contrast solution injection. These examinations give also the opportunity to look for concomitant locations, as well as potential ganglionic or visceral metastasis [4].

The Magnetic Resonance Imaging (MRI) is a very reliable examination that can also detect other localizations despite their small size. The characterization of the tissues is outstanding, especially at the level of the bladder and the vascular axes; MRI is also more adapted than CT in the evaluation of the paragangliomas [8]. In order to enhance the diagnosis and the localization of the paragangliomas, a new imaging method has been recently developed using the iodin-131-MIBG scanning [9]. This examination presents sensitivity of 78% to the adrenal pheochromocytoma, and varies in the range from 67% to 89% when it comes to extra-adrenal localizations.

This examination is more adapted if several localizations are investigated or for the purpose of detection of bone metastasis. However, its high cost is a real limitation. Cystoscopy should be avoided if a bladder pheochromocytoma is suspected, as no prior preparation may have deadly consequences. Through cystoscopy, pheochromocytomas appear as granulated and lobulated lesions with or without ulceration. Similar observations were made for our patient [2,8].

The treatment of the bladder pheochromocytoma is solely surgical and the patient preparation is an essential step, covering a preoperative treatment with α- and β-blocking agents. Surgical resection is the treatment of choice [4]. Due to the multilayer involvement of the bladder wall, open surgery to perform a partial cystectomy is recommended. In the presence of proven metastasis, radical cystectomy with pelvic lymphadenectomy is recommended [1]. Radiotherapy and chemotherapy have limited effectiveness.

A long term follow up with regular biological and clinical examinations is necessary, in order to detect any recurrence or metastasis. From a histological perspective, the usual criteria of malignancy have no ground, but the appearance of a metastasis is a sign of malignancy [4].

Even if 170 cases of bladder pheochromocytoma have been reported so far, only 17 well documented cases are malignant [10]. The prognosis of the bladder pheochromocytoma remains favorable.

## Conclusion

Pheochromocytoma of the bladder is a rare tumor. Its diagnosis is suspected in the presence of paroxysmal hyper blood pressure paired with a bladder tumor. The partial or radical cystectomy remains the treatment of choice in case of malignancy.

## Abbreviations

MRI: Magnetic Resonance Imaging
VMA: vanyl-mandelic acid

## References

[bib-001] Kappers MH, van den Meiracker AH, Alwani RA, Kats E, Baggen MG (2008). Paraganglioma of the urinary bladder. Neth J Med.

[bib-002] Naqiyah I, Rohaizak M, Meah FA, Nazri MJ, Sundram M, Amram AR (2005). Phaeochromocytoma of the urinary bladder. Singapore Med J.

[bib-003] Zimmerman IJ, Biron RE, MacMahan HE (1953). Pheochromocytoma of the 2 urinary bladder. N Engl J Med.

[bib-004] Attyaoui F, Nouira Y (2000). Le phéochromocytome vésical. Progrès en Urologie.

[bib-005] Zidi B, Bouzian A, Ben Hamadi F (1995). Le phéochromocytome extrasurrénalien.bA propos de trois observations et revue de la littérature. Rev Mid Interne.

[bib-006] Goldfarb DA, Novick AC, Bravo EL, Straffon RA, Montie JE, Kay R (1989). Experience with extra-adrenal pheochromocytoma. J Urol.

[bib-007] Messerli FH, Finn M, McPhee AA (1982). Pheochromocytoma of the urinary bladder, systemic hemodynamics and circulating cathecolamine levels. JAMA.

[bib-008] Whalen RK, Althausen AF, Daniels GH (1992). Extra-adrenal pheochromocytoma. J Urol.

[bib-009] Tissaoui K, El Kamel R, Zidi B, Ben Hamadi F, Karray S, Berraies N, Ben Moussa MA (1992). propos d’une observation de phéochromocytome vésical. Prog Urol.

[bib-010] Michel F, Gattegno B, Sicard JF, Roland J, Thibault P (1990). A propos d’une observation de phéochromocytome vésical malin Conduite diagnostique et thérapeutique. Ann Urol.

